# ‘From Defensive Paranoia to …Openness to Outside Scrutiny’: Prison Medical Officers in England and Wales, 1979–86

**DOI:** 10.1017/mdh.2017.77

**Published:** 2018-01

**Authors:** Nicholas Duvall

**Affiliations:** 1 Centre for the History of Medicine, Department of History, University of Warwick, Humanities Building, University Road, Coventry CV4 7AL, UK; 2 Centre for the History of Medicine in Ireland, School of History, University College Dublin, Belfield, Dublin 4, Ireland

**Keywords:** Prison healthcare, Prison Medical Service, Prison Medical Association, Dual loyalty, National Health Service

## Abstract

This article examines how a branch of medicine based within the criminal justice system responded to a society which by the 1970s and 1980s was increasingly critical of the prison system and medical authority. The Prison Medical Service, responsible for the health care of prisoners in England and Wales, was criticised by prison campaigners and doctors alike for being unethical, isolated, secretive, and beholden to the interests of the Home Office rather than those of their patients. While prison doctors responded defensively to criticisms in the 1970s and 1980s, comparing their own standards of practice favourably with those found in the NHS, and arguing that doctors from outside would struggle to cope in the prison environment, by 1985 their attitudes had changed. Giving evidence to a House of Commons committee, prison doctors displayed a much greater willingness to discuss how the prison system made their work more difficult, and expressed a pronounced desire to engage openly with the rest of the profession to address these problems. The change of attitude partly reflects a desire by the Home Secretary William Whitelaw to make the Prison Service more open, and an acceptance of a need for greater accountability in medicine generally. Most important, however, was a greater interest in prison health care and appreciation of the difficulties of prison practice among the wider medical profession, encouraging prison doctors to speak out. This provides a case study of how a professional group could engage openly with criticisms of their work under favourable circumstances.

This article examines how a branch of medicine, based within the prison system in England and Wales, responded to a society which was increasingly critical of medicine and institutions of criminal justice. During the late 1960s and 1970s, several campaign groups emerged representing people from marginalised groups (such as drug users, the mentally ill and prisoners) who found themselves at the sharp end of the attentions of medicine and the criminal justice system. These groups offered a radical critique of institutions and were, in many cases, self-help organisations which engaged in a discourse of civil rights. Release, for example, established in 1967, aimed to provide legal advice to people accused of drug-related offences.^1^ The Mental Patients’ Union, formed in 1971 by psychiatric patients in London, was fiercely critical of ‘mental hospitals and the institution of repressive and manipulative psychiatry’. It questioned the exercising of medical authority over its members’ conditions, and began by seeking to secure rights for patients, including the right to refuse treatment and obtain a second opinion.^2^

Additionally, from the 1960s to the early 1990s, there was a growing appreciation of the status of patients in the National Health Service more broadly. Through participation in Community Health Councils and through the lobbying efforts of patient representative groups, there were increased opportunities for patients to express their views about their own health care. By the 1980s, there was an understanding of and interest in patients’ status as consumers of health care services, with rights and the capacity to exercise choice.^3^

An important element of the increased scrutiny of institutions and the questioning of their representatives’ authority was the attention paid to prisons and prison health care (a context, campaigners noted, where patients’ opportunities for exercising choice were especially curtailed). Commentators agreed that prison conditions were very poor. The criminologists Roy King and Kathleen McDermott characterised the prison system in the 1970s and 1980s as being in ‘ever deepening crisis’, noting severe overcrowding, sanitation problems, and an increase in the time prisoners spent locked in their cells and a decrease in time for work or association.^4^ David Garland has observed that, from this period onwards, retribution and containment were substituted for rehabilitation as the primary objectives of the penal system.^5^ Health care problems were a touchstone, and prison doctors were criticised. Medical officers were seen to be failing to tackle abuses properly. When questioned, after the suppression of a disturbance at Wormwood Scrubs in August 1979, about injuries to prisoners, one doctor appeared to prevaricate about the number who required sutures; the suggestion was also made that suturing was being carried out by underqualified staff.^6^ The pressure group Radical Alternatives to Prison (RAP) accused doctors of prescribing tranquilising drugs ‘in a manner somewhat akin to the way they are used in Russian “prison hospitals” ’.^7^ Another organisation, Women in Prison, condemned doctors for failing to uphold adequate standards of hygiene and for colluding in the harmful punishment of prisoners.^8^

Central to activists’ concerns about the standard of health care in prison was the status of Prison Medical Officers (PMOs) as employees of the Prison Service, which might lead doctors to prioritise the institution’s interests over their patients’. The status of these doctors as civil servants put certain restrictions upon them, such as adherence to the Official Secrets Act, which limited what they could reveal about their work to the outside world.^9^ The careers of those who worked in prisons full-time depended upon the approval of the service, whose main concern was good order and security.^10^ Prisoners’ rights organisations, and others, including from the medical profession, argued that this problem, which present-day scholars of prison medicine term ‘dual loyalty’, could only be solved by removing the administration of inmates’ health care from the prison service entirely, and placing it under the purview of the NHS.^11^ Otherwise, health rights, such as the right to refuse treatment or choose one’s own doctor, would continue to be undermined.

Accounts of the challenges to the authority of medical and social institutions in the 1970s and 1980s have hitherto focused on the voices and actions of groups such as prisoners or patients in psychiatric institutions. The most substantial history of prison medicine in England has been written by Joe Sim. He places prison medicine on a long continuum of ‘control and regulation through medical practice’, and prominently records the responses of campaign groups, such as RAP and the National Prisoners Movement (PROP), and medical and legal critics to the state of prison health care in this period.^12^ However, little attention has been given to the accounts of doctors who worked in the Prison Medical Service (PMS) in this period. This deficit is worth remedying. First, an examination of the responses of prison doctors to their critics provides an example of the changing ways in which a professional group reacted to a critical discourse about their work and the institution in which they operated. In 1979, prison doctors reacted very defensively to a paper by a committee of the Royal College of Psychiatrists which suggested that full-time PMOs were not well placed to look after their patients’ best interests. Yet, in 1985, PMOs spoke openly about the challenges of working within the prison system, and the problems it caused for the doctor–patient relationship, to the House of Commons Social Services Committee, which was carrying out an inquiry into the PMS. The greater willingness of prison doctors to engage with their critics in the 1980s was perhaps a reflection of a broader trend towards greater openness in medicine, and a growing imperative for audit and accountability. Duncan Wilson has shown that by the mid-1980s, the medical profession was more accommodating of advocates of greater accountability, partly because change was seen as inevitable, and partly because engaging could benefit the profession, for example by minimising litigation.^13^ Second, the analysis of PMOs’ viewpoints shows that rather than passively accepting the harmful aspects of prison life, they engaged with questions and concerns about the ‘dual loyalties’ they faced in their work. Indeed, it will be seen that part-time medical officers were among the most critical of the organisation of prison health care.

The Official Secrets Act limited the opportunities for PMOs to speak publicly about their work. Yet, two official inquiries, held in 1979 and 1985–6, represent exceptions to this, and illustrate how prison doctors engaged with questions of dual loyalty. The first example I draw upon is the debate surrounding the Royal College of Psychiatrists’ submission to the 1979 Inquiry into the United Kingdom Prison Services, convened by a judge, Mr Justice May. The College’s evidence, which criticised the fact that prison medicine was not independent of the prison service, elicited a robust, defensive response from several PMOs.^14^ This example is particularly significant because of the controversial place of psychiatry in the debate on prison medicine, relating both to the prescription of psychotropic medication and the difficulty of finding appropriate accommodation for mentally ill prisoners.

The second major example of medical responses to challenges in prison health care is the evidence given by PMOs to the House of Commons Social Services Committee’s inquiry into the PMS, that began in 1985 and reported in 1986. During this inquiry, a range of prison doctors and other interested parties gave evidence to a committee of MPs. In this setting, PMOs reflected more openly on their situation, including on questions related to dual loyalties, and expressed views quite different from those of their colleagues in 1979.^15^ I will consider the reasons for this apparent shift in attitudes on the part of prison doctors, including the impact of new leadership in the PMS, with the appointment in 1983 of a new director, John Kilgour, who accepted the need for change and greater openness in the service, and a broader acknowledgement among the medical profession of the difficulties faced by doctors in prison.

## 1 Background to Prison Medicine

It is worthwhile outlining the context and organisation of prison health care in England and Wales in this period, because it was significantly different from mainstream health care. Many of the criticisms levelled at prison medicine were derived from the particular way it was organised within the Prison Service hierarchy.

Prisoners had a wide range of health care needs, which varied between prisons. Health problems in an overcrowded remand prison differed from those encountered in one housing long-term prisoners. A sizeable number of inmates suffered from mental health problems, with a study of one prison in 1977 finding that an average of 9.25 per cent of the men there needed psychiatric care.^16^ Although PMOs believed that these psychiatrically ill prisoners belonged in NHS hospitals, transferring them there under the Mental Health Act could be difficult.^17^ This led to the suggestion, made by the director of prison medical services, John Kilgour, that ‘some health authorities and psychiatrists [were] refusing to accept their responsibilities and make provision for these difficult patients’.^18^

One study, carried out by Edwin Martin, a general practitioner who practised both in prison and in the community, found that certain conditions, namely schizophrenia, skin infection, minor trauma, abdominal pain and headache, were elevated in prison. He linked these, respectively, to the detention of chronic schizophrenics, overcrowding and restricted bathing opportunities, violence and stress. He noted that prisoners were not permitted to keep household remedies, such as painkillers, in their cells; they had to visit the prison hospital to obtain them.^19^ There were also high incidences of drug and alcohol addiction. Home Office statistics from this period suggested that drug dependence was increasing, from 1003 prisoners reporting dependence in 1979 to 1666 in 1982.^20^

There were many fewer imprisoned women than men in England and Wales. Although very little study of health in women’s prisons had been carried out, Richard Smith reported that doctors at one female institution, HMP Holloway, estimated that ‘women’s prisons had twice as many medical problems as men’s prisons’.^21^ He suggested that ‘female offenders carry a heavy burden of physical and mental abnormality, and there are probably even more of the sad and the mad in women’s prisons that in men’s’.^22^

Attitudes differed among doctors as to whether prisoners constituted a special type of patient. Some suggested that prison doctors needed to recognise ‘that a prisoner may have all sorts of ulterior motives for consulting the doctor’, or noted prisoners’ manipulative qualities.^23^ Diabetic prisoners, for example, sometimes deliberately induced ketoacidosis to bring about removal to the more agreeable surroundings of the prison hospital.^24^ Others preferred to emphasise continuity between primary care inside and outside prison. Martin, for instance, thought that ‘prisoners are a section of the community who are confined within the walls of a prison. … prisoners, on the whole, are not ill, so in the first instance they need primary care’.^25^

Prison health care in England and Wales was not provided by the National Health Service. Instead, it was the responsibility of the Prison Medical Service, which was part of the Prison Service, overseen by the Home Office. Each prison had a hospital, which ranged in size from a small sick-bay to a facility where minor surgery could be performed.^26^ Each prison also had a Prison Medical Officer, a doctor with responsibility for the inmates. The cadre of PMOs consisted of part-time and full-time officers.

The part-time officers were general practitioners who visited the hospital regularly, on a sessional basis, to provide primary care. According to Richard Smith, assistant editor of the *British Medical Journal*, who wrote a series of influential articles about prison medicine between 1983 and 1984, most consultations were with visiting general practitioners. Full-time doctors were concentrated in larger prisons with large numbers of inmates arriving and departing.^27^ As well as providing care for inmates and assessing their condition upon admission, a substantial part of these PMOs’ time was spent compiling psychiatric reports on prisoners for courts and parole boards.^28^ PMOs were also responsible for declaring whether inmates who were in breach of the prison rules were fit to undergo adjudication (a prison disciplinary hearing) and punishment. At that time, the only punishment in use was isolation in a punishment cell.^29^

In male prisons, nursing care was provided by Hospital Officers, discipline officers who had taken a thirteen-week nursing training course. Only a small proportion were state-registered nurses. In female prisons and prison hospitals which provided surgery, nursing was carried out by fully trained nurses.^30^ PMOs and Hospital Officers were employed by the Prison Service, rather than the NHS. They were bound to adhere to civil service codes of conduct, including abiding by the Official Secrets Act, which prohibited them from revealing information about their work in public without permission. The PMS was led by the Director of Prison Medical Services, a doctor who sat on the Prisons Board, the body which oversaw the prison regime.^31^

## 2 Criticisms of Prison Medicine

Critics of the PMS considered its status as part of the prison establishment objectionable. They thought that the medical staff’s dependence on the Prison Service for their livelihood would lead them to put institutional priorities, such as security and good order, ahead of their patients’ best interests. Writing for the Prison Reform Trust, Julian Candy, a psychiatrist who had sat on parole boards, argued that it was ‘inconceivable’ that a doctor whose prospects were wholly in the hands of ‘an enclosed organisation such as the Prison Service, could retain the independence of mind necessary to develop and express cogent criticism of that same organisation’.^32^ Women in Prison argued that, by contrast, community NHS doctors, ‘unpaid and unmuzzled by the Home Office’, would not collude with harmful practices, such as punishment by isolation, ‘and would better protect prisoners from abuse’.^33^

The writing of psychiatric reports about prisoners was a source of conflicts of interest. Candy, who had read many PMOs’ reports to parole boards, noted that they were not always balanced, and that it was rarely specified that consent to write the report had been obtained from the patient (an ethical expectation of NHS psychiatrists).^34^ Smith suggested that even if a doctor made it clear to a prisoner-patient that the contents of a parole or remand consultation would be reported, an ethical dilemma would still exist if ‘the prisoner had seen the doctor the same week at his own request and [had] discussed in that consultation something that he would not want the courts or parole board to know’.^35^

Another source of controversy, related to psychiatry, was the prescription of antipsychotic drugs, such as Largactil (chlorpromazine), to prisoners. From the 1970s onwards, it was alleged that this medication was being used, unethically, to control troublesome behaviour rather than genuine psychiatric illness. The Prison Service maintained that drugs were only used where medically necessary, but this was disputed by prisoners and their supporters. According to one prisoner, quoted in RAP’s magazine in 1979, ‘The Home Office claim that drugs are issued with care is a lie and I feel that this must be constantly reiterated since drug control has undoubtedly taken over (on the whole) from the big stick’.^36^ Others agreed. Tony Whitehead, a psychiatrist and campaigner, analysed Home Office aggregate prescribing data, and concluded that it was likely that drugs were sometimes used to modify behaviour rather than treat illness. He linked this to a general medical tendency to overprescribe, which he thought the closed nature of the prison, with limited external pressure for change, exacerbated.^37^

While still critical of the closed nature of prison medicine, other commentators with experience of visiting prisons suggested that the issue of over-prescription had been exaggerated. The former medical adviser to the Prison Inspectorate, Benjamin Lee, thought that doctors, sensitive to media criticism, were ‘just as likely to withhold adequate doses of psychotropic drugs’, although he admitted the possibility of doctors prescribing to help disciplinary staff achieve ‘a quiet prison’.^38^ In relation to Holloway women’s prison, the prison in which the most psychotropic and hypnotic drugs per head were prescribed, Smith pointed out that many women there would already be taking these drugs when they entered prison.^39^

A more acute problem was the way prison medicine’s structure militated against proper accountability. Smith suggested that scrutiny of prison doctors’ work was too internalised and hierarchical: they reported to the Director of Prison Medical Services, who in turn reported to the director-general of the Prison Service and the Home Office, which was ‘a long internal chain of accountability before outsiders have any say’.^40^ Candy argued that because PMOs often worked in isolation from other doctors, they were subject to little of the informal peer scrutiny which helped to uphold good practice elsewhere.^41^ Campaigners expressed serious concerns about the quality of care in prison. In a joint submission to the Commons Social Services Committee in 1986, PROP, RAP and INQUEST (a group which campaigned about deaths in custody) noted that two recent inquests had determined prisoners died from ‘natural causes aggravated by lack of care’.^42^ In his oral evidence, David Leadbetter of INQUEST repeated an acerbic remark made about one Principal Medical Officer, who held a Diploma in Psychological Medicine, ‘that he really ought to go and ask for his money because he was cheated’.^43^ The medical component of the inspection regime was also deficient. Lee, the inspectorate’s medical adviser, had resigned in frustration in December 1982 because his findings from inspections were filtered by a lay inspector (a seconded governor) who could ‘accept or reject the medical findings in the light of somewhat inconsistent and expedient criteria’.^44^

In a reflection of the broader trend demanding greater patient involvement and choice in the delivery of health care during this period, campaigners argued that establishing the right of inmates to choose their own doctor, namely a GP local to the prison, was crucial to combating the deficit of accountability in prison medicine. As PROP, RAP and INQUEST noted in their 1986 submission:

The crucial difference between prison medicine and general practice is the absence from prisons of the safeguards that protect patients against bad GPs, the right to choose another doctor, and the system of accountability through Family Practitioner Committees, Community Health Councils etc.^45^

During the period covered by this article, therefore, prison medicine was criticised for being subject to conflicts of interest and for unethical prescribing. It was considered unaccountable and sometimes substandard. These views were held not just by prisoners’ rights campaign groups such as RAP, but by fellow doctors, as stated in a *Lancet* news story in 1985: ‘The medical profession has long been concerned by the Prison Medical Service, wherein a bad doctor can practise without scrutiny and as a management pawn’.^46^

## 3 The Royal College of Psychiatrists and the May Inquiry, 1979

In the late 1970s and early 1980s, PMOs engaging with criticisms of the organisation of prison medicine, or responding to allegations of conflicts of interest adopted a defensive attitude. They characterised critics as ill-informed, and the alternative to the PMS, the NHS, as substandard. PMOs themselves countered suggestions that their practice suffered from isolation by emphasising their discipline’s unique skills, which allowed them to flourish in the prison environment. This is shown by several exchanges in the medical press.

In 1979, a submission by the Royal College of Psychiatrists to a judge-led inquiry into the prison system in the United Kingdom was printed in the College’s *Bulletin*. The Committee of Inquiry into the United Kingdom Prison Services, commissioned in 1978 by the Home Secretary Merlyn Rees and chaired by Mr Justice May, a High Court Judge, was mainly concerned with the ability of the prison estate to accommodate a growing population, the control and treatment of offenders and industrial relations with prison staff, but it solicited evidence from a range of parties, including medical bodies.^47^ Prison health care was of clear interest to the College: the preparation of reports on prisoners for the courts, and the fact of the substantial number of inmates suffering from mental health problems, meant that there was a substantial psychiatric component to PMOs’ work. The special committee which prepared the College’s evidence, which included Paul Bowden, a visiting psychiatrist at HMP Brixton, and Brian Cooper, a medical officer at HMP Parkhurst, was very critical of the way in which prison medicine was organised, although they noted that there were serious failings in NHS provision for offenders. In common with other medical critics, they identified the isolation of the PMS from the rest of the profession as the major problem. It was detrimental to doctors’ practical skills, because their experiences were mainly limited to one socio-economic group, and they were unable to continue working with their patients upon their release. The committee also suggested that operating in an isolated environment made PMOs ill-equipped to deal with the ‘ethical problems … raised by the demands of non-medical staff’; they instead denied any conflict of interest.^48^

The College’s solution was that the PMS be absorbed into the NHS, which would then provide prisoners’ medical services. Primary care and basic psychiatric services would be delivered by general practitioners; community physicians would be responsible for public health matters in prisons; and forensic psychiatrists would perform specialist services. Their specialty would be developed ‘to give a service in both prisons and the community’, providing hitherto absent continuity of care. The number of sessions a doctor could spend in prison each week would also be restricted, to ensure they received experience of practice in the community at large.^49^

Several prison doctors provided robust responses to the College’s submission, some of which were printed in the *Bulletin*. While some were measured in their disagreement with the proposals for reform, others were infuriated by the report’s statement that there was ‘a general tendency to reject and scapegoat prisoners so that the services provided for them are often minimal’, which they read as an attack on the PMOs’ characters.^50^

The correspondents’ main objections were to the proposed absorption of the PMS by the NHS, and the provision of services by doctors whose time would be split between the community and the prison. The objectors cast doubt on the likely quality of any NHS services for prisoners, citing existing problems with community health care. David Marjot, a visiting psychotherapist at HMP Wormwood Scrubs, wrote:

To suggest that the NHS is at this time at least capable of taking over the Prison Medical Service is to lose contact with reality. The NHS cannot cope with the task of its own psychiatric and other services; witness the recurrent scandals and problems partly due to an acute shortage of resources.^51^

Rosemary Wool, of HM Young Offender Centre Glen Parva, was similarly caustic: ‘The NHS is floundering – why ask the Prison Medical Service to join a sinking ship!’^52^ Wool did not pretend that living conditions in prison were good, noting problems of old buildings and overcrowding, but she did not consider the NHS able to deliver the better facilities needed by prisoners and staff.

Critics of the College also rejected the suggestion that medical services would be better provided by doctors who only worked in prison part-time. Wool accused the authors of displaying ‘no comprehension of the Prison Medical Officer’s unique role in an establishment’.^53^ Instead, they argued that prison medicine was a specialty which could only be practised effectively full-time. According to Mary Ellis, editor of the *Prison Medical Journal*, it was ‘a highly expert and, in my view, very sophisticated form of medicine which is completely misunderstood by almost everybody in the Health Service’.^54^ The ‘unique role’ described here centred on the ability to deal with the peculiar prison environment, and the supposedly particular qualities of patients, rather than expertise in unusual diseases. Here, the full-time nature of the job was crucial. While outside observers might claim that it led to isolation and the curtailment of professional experience, Wool emphasised the constancy of the full-timer, who maintained ‘close touch with staff and inmates … and so [is] able to act as friend and confidant to both and mediator on occasions’. Part-timers, conversely, found it difficult to pick up the ‘atmosphere’ of a prison because they were only there for a limited time each day.^55^ Ellis suggested that had an experienced PMO been present in HMP Hull in 1976, the serious riot there might have been avoided.^56^ According to another correspondent, R.W.K. Reeves, under the proposed reforms the forensic team tasked with advising on psychiatric matters would be subject to periodic change, and consist of younger, trainee psychiatrists with little prison experience. Advice to discipline and hospital staff as to prisoners’ culpability for breaches of rules, for example, might be inconsistent, to the detriment of staff morale.^57^

Ellis suggested that there was something distinct about dealing with prisoners as a patient group, which required special experience. She stated that they were not ‘ordinary men’ to be handled like others, but liable to manipulate people, including less experienced and part-time doctors. While full-time doctors could develop a relationship of trust with inmates, ‘people who come in and out … on the whole, are not seen as anything other than people to be manipulated’.^58^

Interestingly, the PMOs responding to the College’s evidence did not engage with the question of the conflict of interest for doctors who were employed by the Prison Service, with the exception of Ellis, who saw the issue as a further example of her patients’ potential for mendacity. Regarding the supposed ethical dilemmas raised by the requests of discipline staff, she wrote, ‘In my view, this is a fantasy again which has gained credence by the fact that prisoners are believed and Prison Medical Officers are not’.^59^

Although Ellis was dismissive of the issue of conflicting interests, PMOs did engage with questions of ethics elsewhere. One of the most controversial conflicts concerned the accusation that psychotropic drugs were administered to prisoners to achieve quiescence, rather than to alleviate specific symptoms. J.K. Lotinga, PMO at HMP Wormwood Scrubs, wrote to *The Lancet* in 1977 after an editorial alluded to this practice. Like Ellis, he suggested that spokesmen of prisoners’ rights organisations, ‘themselves ex-prisoners [and] well-known to prison staff’, were making untrue statements to the press. He also went further, stating that principles of medical ethics, surrounding a patient’s right to refuse treatment, were respected: ‘one would not so much as place a stethoscope on the chest of any individual in custody who declined to allow it’.^60^ Stating that patients’ rights were adhered to in prison was also the response to a 1982 article and editorial in *World Medicine*, a magazine aimed at general practitioners, which again alleged that drugs were being prescribed to prisoners to control behaviour.^61^ In this case, J.B. Nunneley, a visiting venereologist to HMP Holloway, replied that according to her own observations and experience, ‘the prisoner’s right to refusal is paramount’. As with the respondents to the College’s evidence, she invoked, as a contrast, the shortcomings of NHS practice, where the existence of patients’ informed consent was questionable: ‘In many hospitals, it is common practice to dish out hypnotics like sweets on the night medicine round’.^62^

Major issues invoked by prison rights campaigners in this period were thus discussed in medical forums. The College was concerned that prisoners, a marginalised group, were receiving an inferior service, and its report raised the issue of whether a prison doctor owed greater loyalty to the Prison Service or the prisoner. Allegations about the misuse of psychotropic drugs were denied by Lotinga and Nunneley, who maintained that the patient’s right to refuse treatment was respected. Ellis, however, responded to the question of conflicts of interest briefly and dismissively. Indeed, the main substance of the discussion around the College’s report concerned the administration of prison health care, focusing on institutional questions, logistics and professional development, rather than inmates’ rights.

The PMOs who responded defensively to the College’s criticisms only represented one group of PMS staff, full-time psychiatrists. Indeed, the contribution of Cooper and Bowden to the College report suggests that opinion was divided even among psychiatrists working with prisoners. The views of part-time PMOs, whose limitations Ellis and Wool alluded to in their letters, would probably have been different, and may have been closer to those expressed in 1985 by the PMOs who gave evidence to the Commons Social Services Committee, discussed below.

## 4 The Prison Medical Association and the Social Services Committee

The Commons Social Services Committee inquiry into the PMS in 1985–6 exposed the views of a broader range of prison doctors than in 1979. These PMOs displayed a much greater willingness to discuss conflicts of interest, the pressures of their employment situation, and the difficulties these caused in their relationships with their patients. They also expressed an eagerness for closer working relationships with the rest of the medical profession and, through their frank participation in the inquiry, the wider public.

The House of Commons Social Services Committee was the parliamentary body responsible for examining the ‘expenditure, administration and policy of the Department of Health and Social Security [and] associated public bodies’. It decided in May 1985, with the agreement of the Home Affairs Committee (which scrutinised the work of the Home Office), to inquire into the PMS in the wake of ‘serious criticisms’ of and ‘alarming stories’ about the Service. Members of the Committee, chaired by Renée Short MP, visited nineteen prisons across the United Kingdom, and took evidence from a wide range of groups. These included: prisoners’ rights and reform organisations, such as RAP, PROP and the Prison Reform Trust; medical bodies, such as the British Medical Association and the Royal Colleges of Psychiatrists and General Practitioners; the PMS leadership, including John Kilgour, the Director of Prison Medical Services; and a delegation from the Prison Medical Association (PMA), a representative body for PMOs.^63^

The PMA was established in 1983 ‘to promote high standards of medical practice in British penal establishments’.^64^ It had no official status with the Home Office, but functioned as a form of self-help organisation for PMOs. One of its first motions was for the institution of ‘a substantial clinical responsibility allowance … which would compensate for the sometimes stressful circumstances of dealing with uncooperative and difficult patients’, as well as raise their earnings to a level comparable to those of general practitioners.^65^ The organisation does not appear to have survived beyond the mid 1980s (according to the obituary of its founder, Dudleigh Topp, it ‘seemed to fall apart after he retired’ in 1984^66^), and it has left little mark upon the historical record beyond letters to the medical and lay press from its chairman, P.A. Trafford, and its evidence to the Social Services Committee in 1985. Yet, this evidence is highly significant, because it shows members’ concerns about the obstacles to good practice which existed in the prison system.

Unlike the doctors who responded to the Royal College of Psychiatrists’ 1979 submission to the May inquiry, the PMA, giving evidence to the Social Services Committee, shared some of the concerns of critics of prison medicine about the position of doctors in the prison establishment and how their role was defined. For example, Women in Prison was very critical of medical participation in the disciplinary process: ‘Doctors performing the function of finding prisoners “fit” are believed to be punitive for sanctioning such barbaric punishment and are held in contempt by many prisoners’.^67^ The PMA likewise argued that their role brought them into conflict with inmates. According to their written evidence, ‘The doctor’s primary responsibility must be to the patient and it is to the detriment of the doctor/patient relationship that the doctor is also seen to be involved in management’.^68^ Trafford stated, ‘we should not be asked to certify a man fit for punishment, which might identify us in the prisoner’s eyes with the selection of somebody for punishment’.^69^ The problem was not solely one of declaring whether a prisoner could withstand punishment, but also of determining whether they understood the adjudication procedure. In Trafford’s view, if, just prior to going before the governor, ‘one has to make some sort of conversation with him to assess that he is in his right mind and able to answer the charge against him, we are seen very much as the initial part of the prosecution, in those circumstances’. His preferred option was that, as in court, inmates be presumed able to answer charges, with the doctor intervening afterwards if necessary. ‘Then [the prisoner] would be seeing that we were intervening on his behalf. After all, the patient should be our first concern, not the Home Office’.^70^

Some of the Association’s concerns were shared by John Kilgour, the Director of Prison Medical Services. While he thought it appropriate for the doctor to determine whether a prisoner was fit to appear before an adjudication committee, he objected to doctors being asked whether an inmate was ‘fit for punishment’, because it gave the appearance of ‘recommending punishment almost’:

To the inmate population it must make them feel that the doctor, aside from looking after his personal mental and physical health, is in fact on the side of the people who are going to punish him.^71^

He argued that the relevant certificate ought to ask the doctor to state any medical reason why cellular confinement should be avoided. Thus, ‘the only time the medical officer needs to say something positive will be on behalf of, protective towards, the inmate, rather than giving a carte blanche for punishment’.^72^

It is to be noted that neither the PMA nor Kilgour were arguing against the practice of confinement as punishment, rather that they ought not be automatically involved when it was inflicted, because it damaged the trust between them and their patients. By contrast, Women in Prison explicitly argued that confinement was harmful.

Causing inmates to distrust them was not the only way in which PMOs’ position within the Prison Service compromised their ability to practise effectively. PMA witnesses argued that their status as Home Office employees, and their place in the prison’s management hierarchy, made their advice on the issues of hygiene and living standards in the prison inefficacious. They recognised that conditions were poor, a result of overcrowding. In their written memorandum, they noted an absence of proper sanitary facilities, the persistence of ‘the degrading act of “slopping out” … an anachronism in 1985’, and instances of pigeon and cockroach infestation. It was the duty of the medical officer to provide the prison’s governor with regular reports on these matters. However, the reports ‘are probably so routine that no account is taken of them’.^73^

This point was reiterated during the doctors’ oral testimony. Trafford, using the example of a doctor complaining about there being no officers to supervise the bath-house, stated, ‘We can say it, but we are talking to the wall very often’. He suggested that because they had been deficient for so long, problems such as the lack of sanitary facilities had become accepted. PMOs’ position within a prison’s management hierarchy allowed their advice to be ignored, and themselves to be cast as complicit: ‘I think that if we were seen in some way as advisers, not as part of the management, it would help. It should not just be said, “Well, you’ve tolerated this for X number of years” ’.^74^ Brian Cooper, also giving evidence, argued that part of the problem was that there were no specific rules, along the lines of Health and Safety at Work regulations, by which the prison service was obliged to provide certain facilities:

They always say [in response to reports] ‘Well, it was worse five years ago’. You see, there are no minimum standards laid down in prisons as regards overcrowding, the number of toilets, the amount of living space, the quality of medical care or anything, so we are really working against a background which is elastic and can be manipulated according to the number of people you have in the system.^75^

The terms of their employment, in particular their status as civil servants, restricted PMOs’ ability to speak publicly about hygiene and other issues, or to correct misconceptions about the nature of prison medicine. All aspects of prison work, including medicine, were covered by the Official Secrets Act, which, according to Edwin Martin, the part-time PMO at Bedford, discouraged prison research and ‘isolated prison doctors from open debate, discussion, and audit of their work’.^76^ Indeed, the Royal College of General Practitioners’ report on prison medicine, submitted to the Social Services Committee, suggested that the report itself represented a criminal act, because of the information it contained.^77^ As for speaking publicly in response to media criticism, Trafford noted that they were ‘bound … by the terms of civil servants who are not allowed to write to the press’, and so were unable to rebut incorrect statements. Trafford said he had himself been censured by the Home Office after writing to *The Times*, although he did not specify about which subject his superiors had objected to his writing.^78^ He had written two letters: the first in October 1983 welcoming the raising of the issue of prison hygiene by the Prison Governors Association, when little attention had been paid to accounts of the problem in PMOs’ hygiene reports; the second in August 1984 arguing that the unavailability of NHS accommodation, rather than prison bureaucracy, was the cause of the long periods spent in custody by mentally ill prisoners on remand.^79^ PMOs’ position as Prison Service employees meant that there was a tangible risk to speaking publicly. Marjorie Hare claimed, in a letter to the *British Medical Journal* in March 1984, that she had been dismissed from her post as a consultant psychiatrist at HMP Grendon after writing an evidently objectionable letter to *The Guardian*.^80^

While the PMOs who responded to the Royal College of Psychiatrists’ report in 1979 emphasised the special experience required to operate successfully in prison, questioning the competence of others to become involved in prison work, the PMA witnesses to the 1985 inquiry expressed a very different view. They sought to build closer bonds with the wider medical profession and with others, such as environmental health officers, relationships which they believed would help tackle the problems they faced within the Prison Service. Because the advice of internal PMOs could be overlooked, it was suggested that outside colleagues could help resolve the situation. With reference to experiences of recommendations being ignored, Cooper suggested that ‘having access to outside bodies who report and look at things are matters that would aid us very much along these lines’.^81^ Likewise, the Association’s written memorandum stated:

It would be logical for the Home Office either to open its doors to local Health Authority Community Physicians and Environmental Health Inspectors or to appoint such officers of its own to ensure that the hygiene of prison establishments falls into line with other public buildings which are not Crown property.^82^

Trafford, noting ‘occasions when medical services are provided under conditions which are far from ideal’, said, ‘I think there is a lot to be said here for opening the doors, letting our colleagues outside – and perhaps not only colleagues but some lay people as well – see the problems of the conditions under which we do work and under which we try to provide a satisfactory service, discussing with them ways in which this could be improved’.^83^ Cooper agreed. Although he was conscious of his patients’ privacy, he envisaged other professionals, especially specialists from the local area, ‘coming in, evaluating and suggesting the work that we do and how it can be improved’. This was something which, he argued, the Prison Inspectorate could not presently achieve, because its medical complement was only one ‘overstrained’ doctor.^84^ Sir Douglas Black, leading the British Medical Association (BMA) delegation, supported this proposal. Visitors could ‘make suggestions for improvement, and perhaps add a little bit of steam to see that suggestions were actually implemented, as sometimes needs doing’. Prison doctors could also raise their own concerns with visiting colleagues.^85^

The PMA witnesses were not the only PMOs to give evidence who advocated greater engagement with the rest of the profession. The Royal College of General Practitioners was represented at the inquiry by two part-time PMOs, Edwin Martin from HMP Bedford and David Jolliffe from HMP Edinburgh, who both also practised in the community. They believed strongly that prison medicine ought to be much more open, inviting external scrutiny of its standards. In their report, they wrote:

The whole work of the prison medical service should be audited. This should not be hidden behind the Official Secrets Act. The audit shold [sic] preferably be done by groups of peers, but it should be seen to be done and the results published. A comparison with medical care in the community would be easier if prison doctors were employed within the National Health Service. … The whole morale of the prison medical service needs to change from defensive paranoia to aggressive investigation, standard setting, and openness to outside scrutiny.^86^

Mirroring the evidence of the PMA, they argued that achieving openness and criticising problems were especially difficult for full-time officers, who were employed by the Home Office. ‘If [the medical officer] causes ripples his career will suffer’.^87^ There was a serious risk of becoming institutionalised, especially when first working in prison. According to Martin, ‘the hospital officers do take you round to begin with, but there is a danger in that because if one is not careful the hospital officers can impose the ethic of the institution on one when one is at a very vulnerable stage’.^88^

Unlike Ellis and Wool in 1979, Martin and Jolliffe argued that there were distinct advantages to part-time practice. It not only meant that high standards of primary care could be brought into prison, but it protected against institutionalisation – the GPs did not consider themselves to be part of the institution. This, Jolliffe said, allowed them to focus more on prisoners’ welfare.^89^ Indeed, they argued that the role of the doctor ought to be further removed from the prison establishment, to facilitate oversight and to place the patients’ needs ahead of the institution’s. They believed that doctors ought not to supervise punishment, and that prison health care ought to be administered by the NHS.^90^ On this last point, they differed from Cooper’s evidence for the PMA; his knowledge of the shortcomings of NHS regional secure units led him to question whether a takeover would lead to better outcomes for prisoner-patients.^91^

It can thus be seen that the 1985–6 Social Services Committee inquiry offered a very different representation of prison medicine than that reflected in the discourse surrounding the May inquiry in 1979. PMOs had gained a more organised voice in the form of the PMA, and they displayed different attitudes. Given an opportunity to speak openly in a public forum, their criticisms of the prison system coincided with some of those of reformers and prisoners’ rights campaigners. The doctors felt their proximity to the system of punishment for disciplinary offences (mandated by the Prison Rules) damaged their relationship with their patients. Their position within the prison management system, coupled with the exemption, under Crown Immunity, of prisons from health and safety standards and regulations, meant that doctors were powerless to remedy the serious problems of the prison environment. The Prison Service could punish any employees who wrote critically to the press, by dismissal or retardation of career progression.

In contrast to the hostility to critics displayed in PMOs’ responses to the 1979 report of the Royal College of Psychiatrists, PMOs in 1985 wanted to enhance links with colleagues working outside who might provide advice or give their recommendations extra clout. Part-timers vocally extolled the merits of practising primary care across the boundary wall, in prison and in the community, and exposing their work to external scrutiny. This enthusiasm for building links with the world outside the prison wall was paralleled by the recommendations made by the Social Services Committee, in their final report, about the treatment of drug addiction among prisoners. Though acknowledged to be a social rather than a medical problem, the committee noted that there was little done to address the psychological causes of addiction, and that in most cases there was no aftercare of drug or alcohol abusers after their release from custody. This was a problem because of both a lack of community facilities and a lack of ambition in the PMS. Noting that there were instances, such as in Staffordshire and Manchester, of prisons working with local NHS services and voluntary groups on addiction, the committee recommended that ‘the Home Office take steps to improve liaison at a local level between prisons and drug and alcohol rehabilitation projects; extend the use of parole for drug and alcohol abusers on condition of attendance at a specialist facility; [and] consider establishing pre-release hostels at selected prisons’.^92^

The reasons for the difference between the views of PMOs in 1979 and 1985 do not lie with any fundamental change in prison working conditions over six years. Such a shift was not identified by the 1985 witnesses. Part of the explanation rests with the differing makeup of those voicing their opinions in 1979 and 1985. The doctors reacting to the Royal College of Psychiatrists’ report in 1979 were, primarily, full-time PMOs seeking to articulate the superiority of their status, whereas there were several part-timers among the prison doctors giving evidence in 1985. This group would have rejected the notion that their visiting status made them less effective within the prison environment; they instead argued that their experience of community practice allowed them to deliver a higher standard of care to their patients in prison. Yet, this does not wholly explain the difference in attitudes. In 1985, full-time PMOs, such as Trafford, were also arguing for greater co-operation with the rest of the medical profession, and criticising the position in which the prison system placed them.

Changes in the attitudes of prison doctors towards external collaboration and scrutiny in this period should be viewed in the wider context of the growing importance of audit in medicine, that is, ‘the review of medical work by medical people’ (the potential of which for prison medicine was hailed in the Royal College of General Practitioners’ submission).^93^ While it was a source of controversy in medicine, audit was formally supported by professional medical bodies.^94^ Duncan Wilson has also identified a growing acceptance among senior members of the profession that external, non-medical oversight of the profession was inevitable, particularly after the Conservative party, which advocated greater accountability of public services, formed a government in 1979.^95^

There was a political imperative for prison medicine to become more open with the change of government in 1979. One of the priorities of William Whitelaw, Home Secretary from 1979 to 1983, had been ‘to open up the prisons to the media and so stimulate public interest and debate as the essential background to remedial action’.^96^ According to Smith, writing in 1983, this had begun, but there were still significant obstacles to those who wanted to produce ‘serious, balanced articles’ about prison, including a dearth of academic research and ‘paranoia’ within the service, especially the PMS. For example, when Smith embarked on the research for his *British Medical Journal* articles, the then Acting Director of Prison Medical Services, Ronald Ingrey-Senn, was suspicious of him, although later his attitude changed and he co-operated with Smith.^97^

Although Ingrey-Senn became more accommodating towards Smith, his replacement as head of the PMS in 1983 also contributed to the observable shift in PMO attitudes. John Kilgour, the new Director of Prison Medical Services, had previously worked at the World Health Organization and the Department for Health and Social Security.^98^ He encouraged PMOs to give evidence to the Social Services Committee, which thanked him for this, and according to Martin, an advocate of openness, Kilgour was much more willing to listen to the concerns of his staff than his predecessor, whom Martin had found ‘extremely difficult to work with’.^99^

Yet, changes in the political priorities of the Home Office and new leadership of the PMS only form part of the explanation for the shift in the participation of prison doctors in public discussion of prison health care. In 1985, circumstances were more propitious for PMOs to engage with other doctors and Members of Parliament in the discussion of prison medicine. Just as some in the wider profession were coming to see the advantages of greater accountability and scrutiny of standards through audit, PMOs speaking in 1985 believed that bringing in their colleagues from outside the Prison Service would help them to address issues such as inadequate sanitation which were currently neglected. That they felt able to express this view is indicative of a medical profession which was itself more willing to engage with its members working in prison, and which was more appreciative of the difficulties facing PMOs in their working environment. For example, the BMA delegation to the Social Services Committee, giving evidence alongside the PMA, was supportive of external doctors working with PMOs, and sympathetic to the fact that ‘the Home Office is not enthusiastic over accepting criticism from employees’.^100^

Richard Smith’s work was important in fostering greater medical interest in the prison world. He embarked on his series of articles, which were later collected in a book, after being struck by the lack of reliable information about health care services in prison, and the deprecatory attitude towards them held by the public and doctors at large: ‘the idea that prison doctors drug prisoners, close their eyes to brutality, identify with prison governors rather than prisoners, and think of prisoners as prisoners first and patients second is deeply rooted’.^101^ Smith produced a thorough and more balanced account of prison medicine and the problems associated with it than previous coverage in publications such as *World Medicine*, which had provoked a hostile reaction. While critical of aspects of the PMS, he was sympathetic to the difficulties faced by PMOs: according to one reviewer, the book showed that ‘[coping] with hunger strikes, self-mutilators, arsonists, malcontents, and hordes of men on sick parade because they have nothing better to do, or cannot otherwise be given an aspirin, is no sinecure’.^102^ Certainly, Kilgour was willing to engage with Smith’s work, which he described as ‘a signal contribution’. He wrote a column in the *British Medical Journal* responding to some of the issues raised by Smith’s series (‘less a riposte than a descriptive commentary on the same problems seen from my unique viewpoint’), and in 1985 he took part in a round-table discussion on fostering further co-operation between the PMS and the NHS with Smith, John Gunn of the Institute of Psychiatry, and Brian Edwards, general manager of Trent Regional Health Authority.^103^ Thus, by the time of the Social Services Committee inquiry, there was already a forum for discussing prison practice within the medical profession. Indeed, such was the significance of Smith’s work that the committee cited his analysis of prison medicine early in their report.^104^

## 5 Conclusions

This article has examined the position of Prison Medical Officers in England in the 1970s and 1980s, and how they perceived themselves, their work and their critics. This was a period when, like that of their counterparts in psychiatric hospitals and the criminal justice system more generally, their authority was being called into question. Prisoners’ groups associated medicine with the punitive aspects of prison life, and accused doctors of prescribing drugs to keep prisoners docile. Other sections of the medical profession saw the PMOs’ place within the prison service hierarchy as problematic, pointing to a potential conflict between patient and institutional interests.

Medical critiques were exemplified in the submission by the Royal College of Psychiatrists to the May inquiry into the UK prison system in 1979. It advocated an NHS takeover of the Prison Medical Service, because Prison Service control was not in patients’ best interests. It also suggested that full-time medical officers were isolated, being exposed only to a limited variety of cases. This prompted a hostile, defensive response from various PMOs. Insulted by the College’s comments, they argued that they in fact possessed a specialist ability to deal with a highly manipulative patient group. They mocked the notion that the NHS would be able to provide a better standard of care for prisoners, especially those with mental health problems. They dismissed the idea, expounded by rights campaigners, that they were ethically compromised, suggesting that campaigners were putting about falsehoods.

By 1985, PMOs displayed a very different attitude. Giving evidence to a parliamentary committee, their testimony was in greater accord with that of their critics. Through the Prison Medical Association, they acknowledged that there were aspects of the prison regime, namely their involvement in certifying prisoners fit for punishment, which damaged the doctor–patient relationship. Their position within the management structure of the prison limited their ability to tackle hygiene problems, because authorities did not act on their recommendations. They were concerned that they would be reprimanded or worse if they criticised their employer publicly. To remedy the situation, they desired to work closer with the rest of the profession, which could both offer advice and exert pressure on prison authorities. Part-time PMOs, influenced by developments in general practice, were eager to adopt systems of auditing clinical work to raise standards.

This pattern of change mirrored a trend within British medicine in the early 1980s of a greater acceptance of oversight, linked to political enthusiasm for accountability of public services. Political expedient during this period can partly explain why PMOs in 1985 were more willing to engage positively with other doctors and openly discuss problems of the prison environment. William Whitelaw, the Home Secretary from 1979–83, had called for openness in the service in general, and John Kilgour, who became Director of Prison Medical Services in 1983, fostered greater engagement. Beyond this, politicians and the wider medical profession were becoming more understanding of the difficulties of working in prison, in part thanks to the journalism of Richard Smith in the *British Medical Journal*.

This account of the diverse and changing attitudes of the doctors who worked in prisons complicates the limited existing historiography of late twentieth-century prison health care, which has previously given little voice to the views of PMOs. While their defensive and arguably security-focused responses in 1979 did not contradict Sim’s interpretation of medicine as a disciplinary tool, it has now been shown that, by the 1980s, many doctors were highly ambivalent about their position in the prison system and the way this compromised their efforts to best help their patients. Prison doctors were among those who participated in critiques of the service. Indeed, the two representatives of the Royal College of General Practitioners at the Social Services Committee inquiry were part-time prison medical officers, which Sim, who cites their highly critical report, does not acknowledge.^105^ In the wider context of the questioning of medical and state authority in this period, this study shows that medical groups who came under critical scrutiny could respond with hostility, defending the status quo, but showed themselves willing to engage with criticisms under the right circumstances.

In spite of the clear enthusiasm expressed by participants in the Social Services Committee hearings in 1985 and 1986 for engagement across prison walls, with a view to improving standards, many of the problems identified by witnesses were to persist. Speaking at a conference in 1992, Dr Vin Chiang, the Head of Medical Services at HMP Brixton, noted that there was a ‘cultural preoccupation with security and control which is entrained into some staff’, which could ‘become especially relevant when staff advise a pliable part-time medical officer who subsequently assumes the role of the managing medical officer’.^106^ A 1996 report by HM Inspectorate of Prisons stated that prison medical staff, in particular nurses, were isolated from their NHS counterparts. Consequently, ‘[t]he constant flow of new ideas, training and development within the National Health Service passes them by’. This problem could only be remedied, the report argued, by placing the medical and nursing staff within ‘mainstream providers of health care’.^107^ In response to this report, a joint working group of Prison Service and NHS Executive Officials, in 1999, recommended that a formal partnership be established between the Prison Service and NHS, to ensure proper integration of services and continuity of care between prison and the community.^108^ In 2006, the responsibility for commissioning health care in English and Welsh prisons was transferred to NHS primary care trusts from the Home Office. According to a Public Health England report, subsequent changes have led to ‘significant improvements in quality of care’.^109^

## Footnotes

1.

Alex Mold , ‘“The Welfare Branch of the Alternative Society?” The Work of Drug Voluntary Organization Release, 1967–1978’, Twentieth Century British History, 17, 1 (2006), 50–73.

2.

Nick Crossley , Contesting Psychiatry: Social Movements in Mental Health (Abingdon: Routledge, 2006) 150. Peer support groups for people coming into oppositional contact with the state continued to be established into the 1980s, for example, Parents Against Injustice, which offered support to parents accused of abusing their children. Jennifer Crane, ‘Painful Times: The Emergence and Campaigning of Parents Against Injustice in 1980s Britain’, *Twentieth Century British History*, 26, 3 (2015), 450–76.10.1093/tcbh/hwv024PMC480423026502666

3.

Glen O’Hara , ‘The Complexities of “Consumerism”: Choice, Collectivism and Participation within Britain’s National Health Service, *c*.1961–*c*.1979’, Social History of Medicine, 26, 2 (2013), 288–304. Alex Mold, ‘Making the Patient-Consumer in Margaret Thatcher’s Britain’, *The Historical Journal*, 54, 2 (2011), 509–28.2477197610.1093/shm/hks062PMC3635502

4.

Roy D. King and Kathleen McDermott , ‘British Prisons 1970–87: The Ever Deepening Crisis’, British Journal of Criminology, 29, 2 (1989), 107–128.

5.

David Garland , The Culture of Control: Crime and Social Order in Contemporary Society (Oxford: Oxford University Press, 2001), 8, 178.

6.

Joe Sim , Medical Power in Prisons: The Prison Medical Service in England 1774–1989 (Milton Keynes: Open University Press, 1990), 108–109.

7.
‘Radical Alternatives to Prison: A Memorandum to the Inquiry into the United Kingdom Prison Services’, *The Abolitionist*, Summer 1979, 12.

8.
1985/86 HC 72-v, House of Commons, Social Services Committee, Session 1985–86, Prison Medical Service, Minutes of Evidence, 12 February 1986, 123, 126.

9.

Richard Smith , Prison Health Care (London: BMA, 1984), 97–98.

10.

Julian Candy , ‘The Relationship of the Prison Medical Service to the National Health Service’, Prison Medicine: Ideas on Health Care in Penal Establishments (London: Prison Reform Trust, 1985), 17.

11.

Jörg Pont , Heino Stöver and Hans Wolff , ‘Dual Loyalty in Prison Health Care’, American Journal of Public Health, 102, 3 (2012), 475–480.2239051010.2105/AJPH.2011.300374PMC3487660

12.
Sim, *op. cit.* (note 6), 103–28, 182. Sim and a co-author, Michael Fitzgerald were previously involved in a legal dispute with three prison doctors over an account of prescribing practices in their book, *British Prisons* ‘Jail Doctors Get Damages over Drug Allegations’, *The Times*, 1 August 1980, 4.

13.

Duncan Wilson , ‘Who Guards the Guardians? Ian Kennedy, Bioethics and the “Ideology of Accountability” in British Medicine’, Social History of Medicine, 25, 1 (2012), 193–211.

14.

K. Rawnsley , ‘The College’s Evidence to the Prison Services Inquiry’, Bulletin of the Royal College of Psychiatrists, 3, 5 (1979), 81–84.

15.
1985/86 HC 72, House of Commons, Third Report from the Social Services Committee, Session 1985–86, Prison Medical Service.

16.

R.A.H. Washbrook , ‘The Psychiatrically Ill Prisoner’, *The Lancet*, 309, 8025 (18 June 1977), 1302.10.1016/s0140-6736(77)91333-268396

17.

G. Robertson and T.C.N. Gibbens , ‘Transfers from Prisons to Local Psychiatric Hospitals under Section 72 of the 1959 Mental Health Act’, *British Medical Journal*, 280, 6226 (24 May 1980), 1263–6.10.1136/bmj.280.6226.1263PMC16015517388498

18.

Brian Edwards , *et al.*, ‘Cooperation Between the Prison Medical Service and the NHS: A Conversation’, *British Medical Journal*, 291, 6510 (14 December 1985), 1698.10.1136/bmj.291.6510.1698PMC14187613000504

19.

Edwin Martin , ‘Comparison of Medical Care in Prison and in General Practice’, *British Medical Journal*, 289, 6450 (13 October 1984), 967–9.10.1136/bmj.289.6450.967PMC14431716435746

20.
Smith, *op. cit.* (note 9), 41.

21.

*Ibid*., 132.

22.

*Ibid*., 128.

23.

*Ibid*, 84, 88; Elizabeth K. McLean, ‘Prison and Humanity’, *The Lancet*, 305, 7905 (1 March 1975), 508.10.1016/s0140-6736(75)92845-746970

24.

G.V. Gill and I.A. MacFarlane , ‘Problems of Diabetics in Prison’, *BMJ*, 298, 6668 (28 January 1989), 222.10.1136/bmj.298.6668.221PMC18355572493867

25.
1985/86 HC 72-iii, House of Commons, Social Services Committee, Session 1985/86, Prison Medical Service, Minutes of Evidence, 4 December 1985, Royal College of General Practitioners, 86.

26.
Smith, *op. cit.* (note 9), 88, 93.

27.

*Ibid.*, 77. The articles first appeared from November 1983 to March 1984, and were collected and updated in book form later in 1984.

28.

*Ibid.*, 86–7.

29.

*Ibid.*, 90–1; UK Statutory Instruments 1964, No. 388, The Prison Rules 1964.

30.
Smith, *op. cit.* (note 9), 113.

31.

*Ibid.*, 76.

32.
Candy, *op. cit.* (note 10), 17.

33.
Social Services Committee, 12 February 1986, *op. cit.* (note 8), 126.

34.
Candy, *op. cit.* (note 10), 18–19.

35.
Smith, *op. cit.* (note 9), 97.

36.
‘The Home Office and the Liquid Cosh’, *The Abolitionist*, January 1979, 6.

37.

Tony Whitehead , ‘The Use of Psychotropic Drugs in Prison’, Prison Medicine: Ideas on Health Care in Penal Establishments (London: Prison Reform Trust, 1985), 71–82.

38.

Benjamin Lee , ‘Free the Prison Doctors Now’, *World Medicine*, 6 August 1983, 21.

39.
Smith, *op. cit.* (note 9), 133.

40.

*Ibid.*, 98.

41.
Candy, *op. cit.* (note 10), 17.

42.
Social Services Committee, 12 February 1986, *op. cit.* (note 8), 133.

43.

*Ibid.*, 143.

44.

Benjamin Lee , ‘On Standing Up and Being Counted’, *The Lancet*, 321, 8336 (4 June 1983), 1269.10.1016/s0140-6736(83)92710-16134051

45.
Social Services Committee, 12 February 1986, *op. cit.* (note 8), 133.

46.
‘Home Office Doctors’, *The Lancet*, 326, 8450 (10 August 1985), 343.

47.

Tim Newburn , Crime and Criminal Justice Policy, 2nd edn (Harlow: Pearson Longman, 2003), 29.

48.
Rawnsley *et al.*, *op. cit.* (note 14), 83.

49.

*Ibid.*, 84.

50.

*Ibid.*, 83.

51.

David Marjot , ‘The Prison Service Inquiry’, Bulletin of the Royal College of Psychiatrists, 3, 8 (1979), 142.

52.

R.J. Wool , ‘The Prison Medical Service’, Bulletin of the Royal College of Psychiatrists, 3, 8 (1979), 142.

53.

*Ibid*.

54.
The National Archives, Kew, United Kingdom Prison Services Committee, Evidence and Papers, Royal College of Psychiatrists, HO 263/30, Letter, Mary Ellis to K. Rawnsley, 16 May 1979.

55.
Wool, *op. cit.* (note 52), 142.

56.
TNA HO 263/30, Ellis to Rawnsley.

57.

R.W.K. Reeves , ‘The Prison Service’, Bulletin of the Royal College of Psychiatrists, 3, 7 (1979), 127.

58.
TNA HO 263/30, Ellis to Rawnsley.

59.

*Ibid*.

60.

J.K. Lotinga , ‘Psychiatry in British Prisons’, *The Lancet*, 310, 8041 (8 October 1977), 765.10.1016/s0140-6736(77)90274-471574

61.

Brian Deer , ‘Doses with their “Porridge” ’, *World Medicine*, 30 October 1982, 28–9; ‘Perverted Medicine’, *World Medicine*, 30 October 1982, 7.

62.

J.B. Nunneley , ‘Prison Medicine’, *World Medicine*, 11 December 1982, 12.

63.
Social Services Committee, Third Report, *op. cit.* (note 15), ii, vii, viii, lxxii–lxxiv.

64.
‘Medical news’, *British Medical Journal*, 286, 6376 (7 May 1983), 1519.

65.
‘Prison Medical Association’, *British Medical Journal*, 288, 6423 (7 April 1984), 1099.

66.
‘Dudleigh Oscar Topp’, *The BMJ*, 353 (11 April 2016), doi:10.1136/bmj.i2068.

67.
Social Services Committee, 12 February 1986, *op. cit.* (note 8), 126.

68.
1985/86 HC 72-ii, House of Commons, Social Services Committee, Session 1985–86, Prison Medical Service, Minutes of Evidence, 27 November 1985, 36.

69.

*Ibid.*, 50.

70.

*Ibid.*, 51.

71.
1985/86 HC 72-i, House of Commons, Social Services Committee, Session 1985–6, Prison Medical Service, Minutes of Evidence, 20 November 1985, 18.

72.

*Ibid.*, 19.

73.
Social Services Committee, 27 November 1985, *op. cit.* (note 68), 37.

74.

*Ibid.*, 51.

75.

*Ibid*.

76.

Edwin Martin , ‘Medical Care in Prisons’, *The Lancet*, 326, 8461 (26 October 1985), 942.10.1016/s0140-6736(85)90868-22865433

77.
Social Services Committee, 4 December 1985, *op. cit.* (note 25), 80.

78.
Social Services Committee, 27 November 1985, *op. cit.* (note 68), 40.

79.

P.A. Trafford , ‘Hygiene in Prisons’, *The Times*, 7 October 1983, 15; P.A. Trafford, ‘Patients in Custody’, *The Times*, 10 August 1984, 11.

80.

Marjorie Hare , ‘Prison Doctor “Ghosted Out” ’, *British Medical Journal*, 288, 6422 (31 March 1984), 1005; Marjorie Hare, ‘Queueing Up for a Cell’, *The Guardian*, 11 February 1984, 12.

81.
Social Services Committee, 27 November 1985, *op. cit.* (note 68), 52.

82.

*Ibid.*, 37.

83.

*Ibid.*, 43.

84.
Social Services Committee, 27 November 1985, *op. cit.* (note 68), 43.

85.

*Ibid.*, 42.

86.
Social Services Committee, 4 December 1985, *op. cit.* (note 25), 83.

87.

*Ibid.*, 80.

88.

*Ibid.*, 86.

89.

*Ibid*.

90.

*Ibid.*, 82.

91.
Social Services Committee, 27 November 1985, *op. cit.* (note 68), 41.

92.
Social Services Committee, Third Report, *op. cit.* (note 15), xxxviii–xl.

93.

Charles D. Shaw , ‘Aspects of Audit: The Background’, *British Medical Journal*, 280, 6226 (24 May 1980), 1256.10.1136/bmj.280.6226.1256PMC16015297388496

94.

D.N. Baron , ‘Can’t Audit? Won’t Audit!’, *British Medical Journal*, 286, 6373 (16 April 1983), 1229–30; Charles D. Shaw, ‘Aspects of Audit: Looking Forward to Audit’, *British Medical Journal*, 280, 6230 (21 June 1980), 1509–11.

95.
Wilson, *op. cit.* (note 13), 201–5.

96.

William Whitelaw , ‘Prisons: No Easy Way Out’, *The Times*, 5 May 1983, 12.

97.
Smith, *op. cit.* (note 9), 5–7.

98.

*Ibid.*, 76.

99.
Social Services Committee, Third Report, *op. cit.* (note 15), vii; Social Services Committee, 4 December 1985, *op. cit.* (note 25), 90.

100.
Social Services Committee, 27 November 1985, *op. cit.* (note 68), 42, 52.

101.
Smith, *op. cit.* (note 9), 7.

102.

D.J. West , ‘Prison Health Care’, *The Lancet*, 324, 8400 (25 August 1984), 438.

103.

John L. Kilgour , ‘The Prison Medical Service in England and Wales: A Commentary from the Director of the Prison Medical Service’, *British Medical Journal*, 288, 6430 (26 May 1984), 1603–5; Edwards *et al.*, *op. cit.* (note 18).10.1136/bmj.288.6430.1603PMC14412486426660

104.
Social Services Committee, Third Report, *op. cit.* (note 15), viii.

105.
Social Services Committee, 4 December 1985, *op. cit.* (note 25), 85; Sim, *op. cit.* (note 6), 121–3.

106.

*Medicine within Prison: The Report of the Conferences which took place on Saturday 27th June 1992 at the London School of Hygiene and Tropical Medicine* ([Dundee]: Physicians for Human Rights (UK), 1992), [13].

107.
Her Majesty’s Chief Inspector of Prisons for England and Wales, *Patient or Prisoner?* (London: Home Office, 1996), 4–5.

108.
Joint Prison Service and National Health Service Executive Working Group, *The Future Organisation of Prison Health Care* (Leeds: NHS Executive, 1999).

109.

*Rapid Review of Evidence of the Impact on Health Outcomes of NHS Commissioned Health Services for People in Secure and Detained Settings to Inform Future Health Interventions and Prioritisation in England* (London: Public Health England, 2016), 8.

